# Supplementary material for: WordCommentsAnalyzer: A windows software tool for qualitative research
Source: F1000Res. 2018 Sep 4;7:536. Originally published 2018 May 3. [Version 2] doi: 10.12688/f1000research.14819.2 (PMC6137411; doi:10.12688/f1000research.14819.2)
Supplement: Supplementary file 3 [file f1000research-7-17614-s0002.tgz › b2a79496-4ada-47c4-9682-a99ce867836d.zip!/Eight Iranian Health Journals/Yafteh _58.docx]

© Yafteh  . This work is licensed under CC BY 4.0 (https://creativecommons.org/licenses/by/4.0/).

Volume & Issue: دوره 6 - شماره 3

Title: روش درمانی جدید استئوآرتریت (مقاله مروری)

http://yafte.lums.ac.ir/article-1-926-fa.html

گلوکزآمین وکوندروتین سولفات اجزای تشکیل دهنده غضروف مفصلی هستند و درخصوصیات فیزیولوژیک ومکانیکی این بافت نقش دارند. گلوکز آمین به عنوان ماده پیش ساز واحد دی ساکاریدی درمولکول گلوکز آمینوگلیکان غضروف نقش مهمی دارد. استفاده از گلوکزآمین وکوندروتین سولفات جهت درمان علامتی استئوآرتریت مورد بحث است ودلایل موافق ومخالف فراوان دارد. صنعت تولید این مواد دارویی طبیعی یک پدیده غیر قابل تنظیم است. این محصولات تحت آزمایش دقیق قرار نگرفته اند؛ کیفیت پایین دارند واغلب جزء فعالی در ترکیب آنها وجود ندارد. با این حال در ارزیابی اثر این مواد طبیعی در محیط مطمئن مصنوعی In vitro و In Vivo (حیوانات آزمایشگاهی وانسان)، شواهد کلینیکی کارآیی ومنظم بودن این محصولات را نشان داده است. این محصولات می توانند جایگاه مهمی در درمان غیر جراحی استئوآرتریت داشته باشند. از جمله این محصولات می‌توان به گلوکز آمین و کوندروتین سولفات اشاره کرد که مدارک متعدد از جمله بیوشیمی کشت بافت مدلهای حیوانی و انسانی آرتریت و شواهد رادیولوژیک، نقش این دو ماده را در درمان استئوآرتریت و جلوگیری از عوارض آن تأئید کرده است. بعد از اینکه تشخیص استئوآرتریت مسجل شد این دو ماده طبیعی نقش خط اول درمان برای بهبود علامتی بیماری و نیز پتانسیل تغییر ماهیت و ساختار بیماری را دارند. گلوکز آمین باید با دوز 400 میلی گرم روزانه داخل عضلانی و یا 1500 میلی گرم خوراکی حداقل برای یک ماه تجویز شود.

Title: تعیین جنسیت به روش Nested-PCRبا استفاده از ژن آمیلوژنین

http://yafte.lums.ac.ir/article-1-928-fa.html

مقدمه: تعیین جنسیت گذشته از اینکه از نظر والدین دارای ارزش خاصی است، در بیماریهای وابسته به جنس نیز از اهمیت زیادی برخوردار است. هدف از این مطالعه تعیین جنسیت جنین در موارد مشکوک به بیماریهای مغلوب وابسته به X در سه ماهه اول بارداری به روش مولکولی بود. مواد و روشها: بعد از جمع آوری74 نمونه از پرزهای کوریونیک (CV) و جدا کردن آنها از نمونه های مادری، DNA آنها استخراج شد و پس از انجام واکنش زنجیره ای پلیمر از (PCR)، جنسیت آنها تعیین شد. همچنین بعد از افزایش حساسیت، سیستم قادر به تعیین جنسیت یک سلول بیوپسی شده از جنین بود.آنالیز آماری با استفاده از آزمون دقیق فیشر انجام گرفت. یافته ها: جنسیت نمونه های CV بوسیله PCR تعیین شد. نتایج آن تعداد از نمونه هایی که موفق به ارتباط با خانواده های آنها شدیم، با جنسیت جنینهای متولد شده یکسان بود(p<0.001). بعد از افزایش حساسیت سیستم، جنسیت 13 نمونه جنینی که در مراحل مختلف سلولی بودند تعیین شد. همچنین جنسیت دو نمونه تخمک لقاح نیافته(سلول هاپلوئید) و یک تخمک بارور شده؛ ولی تقسیم نشده(سلول دیپلوئید)، تعیین شد. نتیجه گیری: تعیین جنسیت جنین قبل از تولد در سه ماهه اول بارداری، از تولد کودکان بیمار جلوگیری کرده و همچنین از ارتباط عاطفی مادر با جنین در موارد سقط می کاهد. همچنین در موارد لقاح آزمایشگاهی(IVF) با شناسایی جنس جنین و انتقال جنین مونث به مادر در بیماریهای مغلوب وابسته به جنس می تواند کاربرد داشته باشد. کاربرد دیگر آن در کنترل جمعیت جوامع در مواردی است که زوجها تمایل به داشتن جنس خاصی از کودک را دارند. سیستم مولکولی اپتیمایز شده فوق که بر اساس ژن آمیلوژنین طراحی گردید، می تواند جنسیت را در نمونه های خون، CV و در مرحله تک سلولی IVF با حساسیت و ویژگی بالا تعیین نماید.

Title: تعیین فراوانی آلودگی لیستریایی در محصولات لبنی ارسالی به آزمایشگاه اداره نظارت بر مواد غذایی و بهداشتی استان لرستان و تعیین الگوی مقاومت آنتی بیوتیکی

http://yafte.lums.ac.ir/article-1-929-fa.html

مقدمه: لیستریا منوسایتوژنز به عنوان یک پاتوژن جدی انتقال یابنده از طریق مواد غذایی شناخته شده است. مشخص شده است که مواد لبنی ممکن است منبع سویه های لیستریا باشند که منجر به لیستریوزیس انسانی شود. هدف از انجام این مطالعه، تعیین میزان آلودگی لیستریایی از محصولات لبنی ارسالی به آزمایشگاه اداره نظارت بر مواد غذایی و بهداشتی لرستان بود. مواد و روشها: در این مطالعه توصیفی 720 نمونه مواد لبنی مختلف ارسالی به آزمایشگاه کنترل مواد غذایی استان لرستان از نظر وجود گونه های لیستریا مورد بررسی قرار گرفت. یک سری تستهایی شامل تولید کاتالاز، CAMP، وگز پروسکوئرVP))، همولیز بتا و تخمیر قندهایی مانند گزیلوز و مانیتول برای تشخیص لیستریا به کار رفت و در نهایت تست آنتی بیوگرام برای تعیین مقاومت آنتی بیوتیکی انجام پذیرفت. یافته ها: لیستریا منوسایتوژنز در 72/9% نمونه ها و لیستریا اینوکوا و لیستریا سیلیگری به ترتیب در 83/5% و 11/1% نمونه ها یافت شد.گونه های دیگر لیستریا در این بررسی ایزوله نگردید. در تست آنتی بیوگرام همه ایزوله ها به پنی سیلین، آمپی سیلین، کوتریموکسازول و جنتامایسین حساس بود و 5 ایزوله لیستریا منوسایتوژنز به اریترومایسین مقاوم بود. نتیجه گیری: با توجه به اینکه مواد لبنی آماده مصرف بوده و امکان آلودگی آنها با گونه های لیستریا زیاد می باشد لذا جداسازی و تشخیص این باکتری در مواد لبنی حائز اهمیت است.

Title: بررسی تغییرات برخی پارامترهای متابولیک سرم ناشی از دپو مدروکسی پروژسترون استات

http://yafte.lums.ac.ir/article-1-930-fa.html

مقدمه: یکی از راههای پیشگیری از بارداری، استفاده از مدروکسی پروژسترون استات است که در آن فقط از پروژسترون استفاده شده و برای مدت چندین ماه از وقوع تخمک گذاری جلوگیری می کند. هدف از این مطالعه بررسی تغییرات برخی لیپیدها و لیپوپروتئین های سرم بعد از 3 و 6 ماه مصرف دپو مدروکسی پروژسترون استات (DMPA) در زنان داوطلب بود. مواد و روشها: در این مطالعه پارامترهای تری گلیسرید، کلسترول، HDL –کلسترول و LDL-کلسترول بیست و یک نفر از داوطلبانی که برای بار اول جهت تزریق DMPA به درمانگاه شهدای همدان مراجعه کرده بودند، هر سه ماه یکبار اندازه گیری شده و نتایج بدست آمده با روش آماری Repeated Measurnment ANOVA و نرم افزارآماری SPSS مورد تجزیه و تحلیل قرارگرفت. یافته ها: نتایج بدست آمده نشان داد طی شش ماه مصرف DMPA مقدار کلسترول و LDL-کلسترول کاهش (p<0.001) و HDL-کلسترول افزایش (p=0.045) معنی داری یافته و تغییری در مقدار تری گلیسرید از نظر آماری مشاهده نگردید. نتیجه گیری: بنابر نتایج حاصل از این مطالعه می توان نتیجه گرفت خطر بروز تصلب شرائین در استفاده کنندگان DMPA وجود ندارد، با این حال ارزیابی سالانه الگوی لیپیدی در زنان مصرف کننده DMPA به مدت طولانی پیشنهاد می شود

Title: بررسی اثر زهر کژدم Hemiscorpius lepturus در تغییرات پاتولوژیک اندام های رت

http://yafte.lums.ac.ir/article-1-931-fa.html

مقدمه: کژدم گادیم Hemiscorpius lepturus (همیسکورپیوس لپتروس) یکی از کژدم‌های خطرناک دنیا و ایران است. این کژدم در استانهای جنوبی به ویژه در خوزستان مسئول گزش تعداد زیادی از افراد بوده که پی‌آمد آن عوارض خطرناک و طولانی و گاهی مرگ است. باتوجه به این که مطالعه اثرات زهر این کژدم در حیوانات آزمایشگاهی می‌تواند، مکانیسم عمل زهر را روشن نموده و کمک زیادی به درمان کژدم زدگان نماید لذا بررسی اثر زهر کژدم گادیم درتغییرات پاتولوژیک اندام های رت انجام شد. مواد و روشها: این مطالعه تجربی بر روی 101 رت از یک نژاد و سن تقریبی 3-2 ماهه و وزن 250-200 گرم صورت پذیرفت. رت‌ها بطور تصادفی به دو گروه شاهد و تجربی تقسیم شدند. به گروه شاهد 1/0 سی‌سی سرم فیزیولوژی و گروه مورد 1/0 سی سی سرم فیزیولوژی که حاوی یک میکرولیتر زهر تازه کژدم گادیم بود در پوست تزریق گردید. سپس تغییرات موضعی و یا زخم با گروه شاهد قبل و بعد از تزریق با آزمون‌های آماری Fisher exact test مورد تحلیل قرار گرفت. بافت کبد، کلیه، طحال در آتوپسی برداشت شد و جهت تشخیص تغییرات پاتولوژیک با میکروسکوپ مورد بررسی و مقایسه قرار گرفت. یافته ها: نتایج پژوهش نشان داد که تزریق زهر کژدم گادیم در 55 درصد موارد در پوست رت موجب زخم گردیده است. تغییرات پاتولوژیک در اثر تزریق زهر این کژدم در کبد به میزان 6/70 درصد، در کلیه 3/35 درصد و در طحال به میزان 4/27 درصد رخ داده است. تغییرات پاتولوژیک در این اندامها شامل خونریزی، احتقان، نکروز با درجه‌های شدید تا ملایم بود. نتیجه گیری: زهر کژدم گادیم در ایجاد آسیب پوستی یا زخم، تغییرات پاتولوژیک در کبد، کلیه و طحال رت، نقش دارد. بررسی ضایعات پوستی و تغییرات پاتولوژیک در اندام‌های مختلف در موارد انسانی، به صورت بیوپسی و آتوپسی موجب شناخت اثرات این زهر در انسان و در نتیجه درمان مناسب‌تر مصدومین می شود.

Title: مقایسه تاَثیر دو روش انحراف فکر با استفاده از موسیقی و تکنیک تنفس منظم

http://yafte.lums.ac.ir/article-1-932-fa.html

مقدمه: ترس از درد و صدمه یکی از مهمترین مشکلات کودکان بیمار است و در صورتیکه هنگام اقدامات تشخیصی و درمانی دردناک از روشهای تسکین دهنده مناسب استفاده نشود، این امر نتایج منفی قابل توجهی را به بار خواهد آورد. این کارآزمایی بالینی به منظور تعیین و مقایسه تاَثیر دو روش انحراف فکر با استفاده از موسیقی و تکنیک تنفس منظم هی-هو بر شدت درد ناشی از رگ گیری در کودکان 12-6 ساله انجام شده است. مواد و روشها: 30 کودک مبتلا به تالاسمی بر اساس مشخصات واحدهای مورد پژوهش از بین کودکان مراجعه کننده به بیمارستان شهید مدنی خرم آباد به روش نمونه گیری در دسترس ومبتنی بر هدف انتخاب شدند، از پرسشنامه مشخصات فردی، مقیاس خودگزارشی اوشر و مقیاس رفتاری چئوپس جهت جمع آوری اطلاعات استفاده شد. هر یک از نمونه ها در سه مرحله یعنی در سه مراجعه متوالی جهت ترانسفوزیون مورد بررسی قرار گرفتند.در مراجعه اول رگ گیری به روش معمول یعنی بدون استفاده از انحراف فکر،در مراجعه دوم با استفاده از موسیقی جهت ایجاد انحراف فکر و در مراجعه سوم با استفاده از تکنیک تنفس منظم هی-هو جهت ایجاد انحراف فکر،اجرا شد.در هر سه مراجعه شدت درد رگ گیری با استفاده از معیار چئوپس حین رگ گیری و معیار اوشر 5-3 دقیقه بعد از رگ گیری تعیین شد. یافته ها: نتایج نشان داد که براساس هر دو معیار، در روش معمول شدت درد رگ گیری، متوسط بود؛ اما در روشهای استفاده از موسیقی و تکنیک تنفس منظم هی-هو شدت درد خفیف بود.همچنین مقایسه شدت درد رگ گیری به سه روش فوق نشان داد که هر دو روش موسیقی و تنفس منظم هی-هو شدت درد رگ گیری کمتری نسبت به روش معمول داشتند (0005/0P<) و در این میان موسیقی دارای کمترین شدت درد بود. نتیجه گیری: پیشنهاد می شود پرستاران ازاین روش تسکین درد جهت مراقبت ازکودکانی که مکررا ًتحت اقدامات درمانی و تشخیصی کوتاه مدت قرار می گیرند استفاده کنند. به این ترتیب از اثرات منفی درد بر کودکان جلوگیری و همکاری آنها را در انجام اقدامات درمانی بیشتر نموده و باعث بهبود کیفیت زندگی و رشد و تکامل کودکان بیمار شوند.

Title: شیوع ترماتودهای کبدی مشترک بین انسان و دام در دامهای کشتار شده و رنگ آمیزی آنها

http://yafte.lums.ac.ir/article-1-933-fa.html

مقدمه: ترماتودهای کبدی، انگل مشترک انسان ودام هستند که دردامها موجب خسارات اقتصادی فراوان می شوند. انسان به عنوان میزبان اصلی انگل در مسیر آلودگی قرار می گیرد و ضایعات کبدی، مخاطرات بهداشتی در جوامع انسانی به بار می آورد. هدف از این پژوهش، بررسی شیوع آلودگی دام ها به تروماتودهای کبدی بر اساس نوع دام، جنس دام واکسیناسیون تهیه لام های آموزشی از ترماتودهای کبدی بود. مواد و روشها: این پژوهش به صورت توصیفی ـ مقطعی انجام گرفت و جامعه مورد مطالعه دامهای کشتار شده در کشتارگاه خرم آباد بوده که کبد2912 دام مورد بررسی قرار گرفت. پس از تعیین نوع آلودگی دام، انگل ها از کبد دام جدا شد و رنگ آمیزی شدند. یافته ها: میزان آلودگی در بز، گوسفند و گاو به ترماتودهای کبدی به ترتیب 6/8، 2/8 و 9/7 درصد بوده، بیشترین میزان آلودگی دام ها مربوط به دیکروسولیوم بوده و همچنین 3/9 درصد دام های ماده و 8 درصد دام های نر آلوده بوداند. نتیجه گیری: با توجه به میزان شیوع آلودگی دام ها به ترماتودهای کبدی و زیانهای اقتصادی فراوان ناشی از آنها لازم است جهت کاهش آلودگی در دام ها، دامپروری به سمت صنعتی شدن سوق داده شود و از علوفه بهداشتی و عاری از آلودگی جهت تغذیه دام ها استفاده گردد.

Title: اثر ترنجبین خوراکی بر کاهش زردی فیزیولوژیک نوزادان

http://yafte.lums.ac.ir/article-1-934-fa.html

مقدمه: هیپربیلی روبینمی یک مشکل شایع و اکثرا خوش خیم نوزادی است که در 80% نوزادهای نارس و 60% نوزادان رسیده رخ می دهد. از روش های درمان زردی، فتوتراپی، تعویض خون و تجویز برخی مواد خوراکی از قبیل شارکول، کلستیرامین و آگار است که با ممانعت از جذب روده ای بیلی روبین سطح سرمی آن را می کاهند. در فرهنگ بومی ایران و بویژه در استان لرستان از ترنجبین به عنوان کاهنده زردی نوزاد به کرات استفاده می شود. هدف این تحقیق بررسی اثر ترنجبین خوراکی در کاستن زردی نوزادان بود. مواد و روشها: این مطالعه به صورت کارآزمایی بالینی در نوزادان رسیده با وزن بالای 2500 کیلوگرم و بدون هرگونه فاکتور خطر انجام شد. به گروه مورد (41 نوزاد) cc/kg1 هر 12 ساعت، سوسپانسیون 30 گرم درصد ترنجبین خوراکی علاوه بر فتوتراپی تجویز و در گروه شاهد فتوتراپی به تنهایی انجام شد. سطح بیلی روبین توتال و مستقیم هر 24 ساعت اندازه گیری شد. نتایج با استفاده از نرم افزار SPSS و آزمون های آنالیز واریانس یک طرفه و آزمون t تجزیه و تحلیل شد. یافته ها: آزمون t نشان داد بین طول مدت بستری دو گروه تفاوت معنی داری نداشته است. همچنین بیلی روبین توتال و مستقیم دو گروه بعد از 24 ساعت تفاوت معنی داری نداشته است؛ ولی بیلی روبین مستقیم 48 ساعت پس از شروع ترنجبین به طور معنی داری پایین تر از گروه شاهد بود (014/0p=). نتیجه گیری: علیرغم باورهای بومی مبنی بر تاثیر ترنجبین در کاستن زردی نوزادی تجویز این ماده اثر قابل توجهی در درمان زردی نداشته و به عنوان درمان زردی پیشنهاد نمی شود.

Title: گزارش یک مورد حالب و لگنچه دوشاخه(Bifid ureter and renal pelvis)

http://yafte.lums.ac.ir/article-1-935-fa.html

مقدمه: حالب دوگانه یکی از ناهنجاریهای مجاری ادراری است که ممکن است بصورت کامل یا ناکامل پدیدار گردد. حالب دو شاخه ناکامل تحت عنوان حالبBifid معروف است. این ناهنجاری به علت اختلال در تکامل جوانه حالبی که برخاسته از مجرای مزونفریک است، در حدود هفته پنجم زندگی داخل رحمی ایجاد می گردد؛ به طوریکه جوانه حالبی (Uretric Bud) بداخل بافت متانفروز نفوذ می کند؛ اما این جوانه حالبی اگر قبل از نفوذ به درون بافت متانفریک به علت اختلال تقسیم شود حالب دوگانه را ایجاد می نماید که دارای یک دهانه خروجی به درون مثانه می باشد. حالب دو شاخه که در گذشته هم دیده شده با دیگر ناهنجاریهای مادرزادی از جمله سندروم Goltz همراه بوده است. مطالعات اخیر در سال 2000 روی تصاویر Urogram فقط 3% حالب دو شاخه را گزارش کرده است و در کتاب مرجع Grant's دو شاخه شدن حالب و لگنچه به میزان 5 صدم درصد گزارش شده است. معرفی مورد: گزارش حاضر مربوط به جسد مرد 65 ساله ای است که در گروه آناتومی دانشکده پزشکی برای دانشجویان پزشکی مورد تشریح قرار گرفت. پس از تشریح حفره شکم، مشاهده شد که علاوه بر بزرگ بودن کبد و طحال و قطور بودن وریدهای کلیوی و بزرگ سیاهرگ زیرین، هر دو کلیه هم بطور غیر طبیعی بزرگ و حالب سمت چپ تا لبه لگن حقیقی دو شاخه شده و بعد از آن بهم پیوسته اند؛ اما در کلیه سمت راست فقط لگنچه دو شاخه شده وحالب از قطب تحتانی کلیه به پایین مسیر طبیعی خود را پیموده است. نتیجه گیری: با توجه به اکثر تحقیقات بعمل آمده این گونه موارد همراه با ناهنجاری بوده اند؛ ولی گزارش حاضر با دیگر ناهنجاریهای مادرزادی همراه نبوده است. البته علل رویان شناسی این امر و تشکیل حالب دو شاخه مورد بحث قرار گرفته است.
